# Scorpionism by *Tityus silvestris* in eastern Brazilian Amazon

**DOI:** 10.1186/s40409-016-0079-2

**Published:** 2016-08-26

**Authors:** Johne Souza Coelho, Edna Aoba Yassui Ishikawa, Paulo Roberto Silva Garcez dos Santos, Pedro Pereira de Oliveira Pardal

**Affiliations:** 1 Postgraduate Program in Tropical Diseases, Center of Tropical Medicine, Federal University of Pará (UFPA), Belém, PA Brazil; 2 Laboratory of Medical Entomology and Venomous Animals, Center of Tropical Medicine, Federal University of Pará (UFPA), Av. Generalíssimo Deodoro, 92, Umarizal, Belém, 66055-240 Pará Brazil

**Keywords:** Scorpion sting, Scorpionism, *Tityus silvestris*, Envenomation, Eastern Brazilian Amazon

## Abstract

**Background:**

Scorpionism is a serious public health problem in Brazil. Although cases of envenomation by scorpions are frequent in Brazil, *Tityus silvestris* – found throughout the Amazon region – is considered of minor medical significance and with only a few descriptions in the literature. This article aims to describe for the first time the epidemiological characteristics and clinical manifestations of scorpion stings by *T. silvestris* that occurred in eastern Brazilian Amazon.

**Methods:**

A prospective and observational study was carried out on 13 confirmed cases of *T. silvestris* envenomation registered from 2007 to 2011 in the cities of Belém and Ananindeua, Pará state, Brazil.

**Results:**

The stings occurred mainly during daytime, at domiciliary environment, and the scorpions were found in clothing, fruits or vegetables. Envenomation was more frequent in the age group between 21 and 30 years old, upper limbs were more affected and medical aid was usually provided within two hours. Men and women were equally affected. Regarding severity, ten patients were classified as Class I and three patients as Class II according to the Scorpion Consensus Expert Group. Local manifestations were present in all patients, being pain the most common symptom. Mild systemic manifestations including nausea, vomiting, somnolence, malaise and prostration were observed in three victims. Symptomatic treatment of pain was offered to all patients, and only one received specific antivenom. All victims had a favorable outcome.

**Conclusions:**

To the best of our knowledge, this study is the first to report the systemic symptomatology of envenomation by *T. silvestris* in the Brazilian Amazon, highlighting the medical relevance of the species in this region. Further research on the venom and clinical manifestations of envenomation by *T. silvestris* should be conducted in order to verify the relevance of this species to public health.

## Background

Scorpion envenomation was considered by the World Health Organization a neglected public health issue [1]. According to Chippaux and Goyffon [2], there are about one million and two hundred thousand cases of envenomation worldwide annually. In Brazil, it constitutes a public health problem [3]. Scorpion stings are among the most frequent causes of envenomation in Brazil, responsible for 30 % of the deaths by this cause in the country [4].

Although in Brazil there are about 160 scorpion species described, only *Tityus serrulatus*, *T. bahiensis*, *T. stigmurus* and *T. obscurus* are considered medically important. Therefore, the species from the Amazon region *T. metuendus*, *T. silvestris* and *Rhopalurus* are of minor medical significance [5].

*T. silvestris* are among the small scorpions of the family Buthidae in Brazil. They are yellow with scattered dark spots and their body size (in adult specimens) reaches 25 to 45 mm. These scorpions present distinct sexual dimorphism and broad distribution in French Guiana and in the Brazilian Amazon (mainly Amazonas and Pará states) [6].

The symptomatology and severity of scorpion envenomation depends on the species, the amount of inoculated venom and the chemical mediators released. A classification of clinical consequences of scorpion stings was created by an international group of experts [7]. However, studies on the envenomation by *T. silvestris* are still scarce. For example, Martins et al. [8] reported four cases and Asano et al. [9] only two, all of them classified as Class I of severity [7]. Because of this lack of information, the present study aims to describe, for the first time, the epidemiological characteristics and the clinical manifestations of scorpionism by *T. silvestris* that occurred in Pará state, eastern Brazilian Amazon.

## Methods

**Fig. 1:**
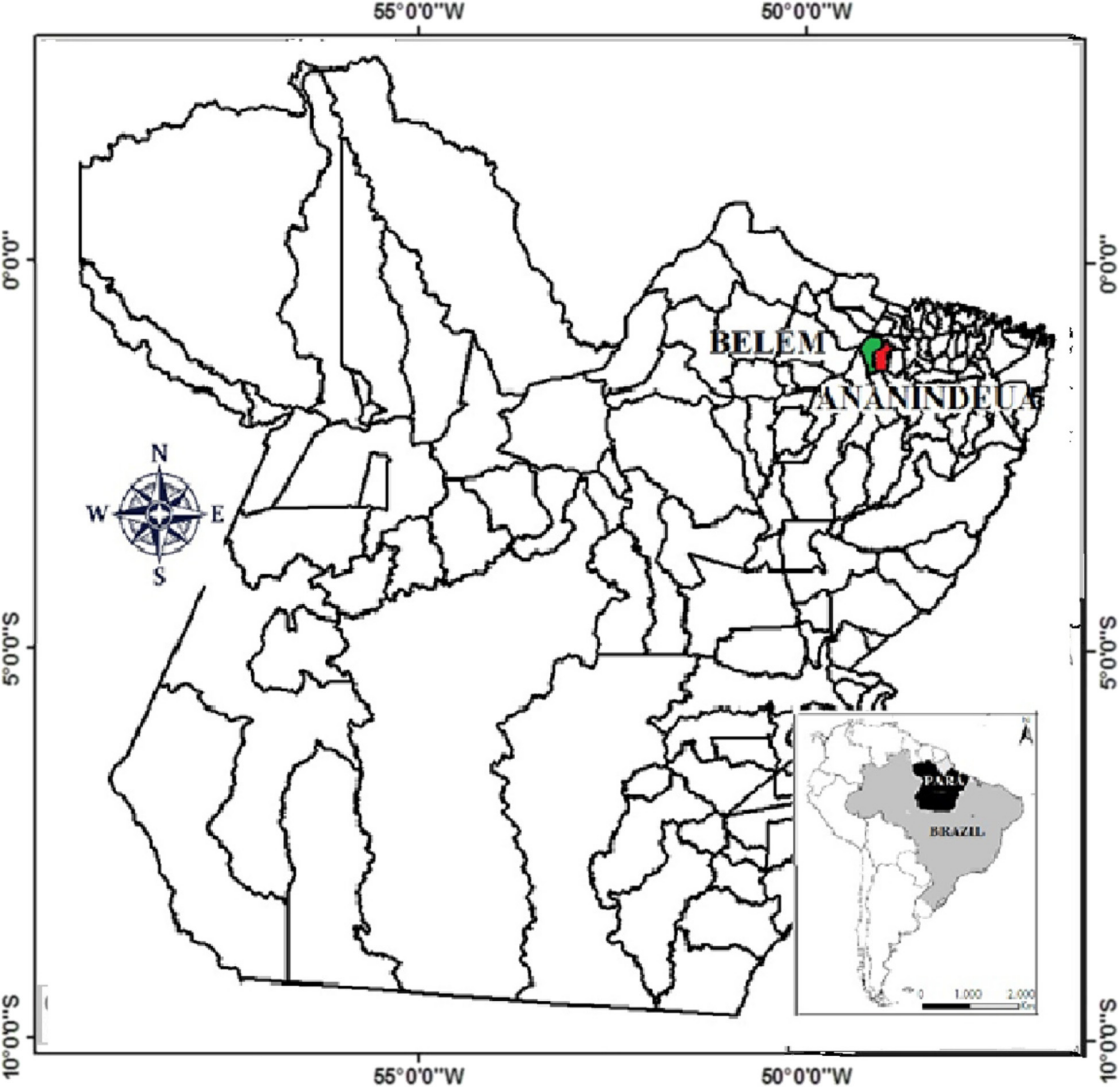
Study areas in Pará state, northern Brazil: Belém (green) and Ananindeua (red) cities

The present study consisted of a prospective and observational analysis based on the records, from 2007 to 2011, of patients envenomed by *T. silvestris* in the cities of Belém (01° 27' 21" S e 48° 30' 16" W) and Ananindeua (01° 21’ 58”S e 48° 22’ 22” W), Pará state, eastern Amazon (Fig. 1). Belém is capital of the state with an area of 1,059.402 km^2^ and 1,432.844 inhabitants. Ananindeua is located at Belém metropolitan area, with an area of 190,452 km^2^ and 499,776 inhabitants [10]. Both cities are surrounded by tropical forests. The climate in these areas is hot and humid, with average annual temperature ranging between 22 ° C and 34 ° C.

### Patients

Thirteen victims of *T. silvestris* were part of this study. All of them voluntarily sought hospital care and confirmed the envenomation providing the specimens to the medical staff. The animals were identified at the Laboratory of Medical Entomology and Venomous Animals, which is part of the Center of Tropical Medicine, Federal University of Pará, Brazil.

Among the available variables, the present study took into account the following data related to the stings: gender and age of the victim, time to medical care, sting site, local and systemic symptoms, severity of envenomation and treatment.

The severity of the scorpion stings was organized based on the classification developed by the Scorpion Consensus Expert Group [7]:

- Class 0 – dry sting or asymptomatic patients.
- Class I – envenomation with manifestations only at the bite site.
- Class II – envenomation with minor systemic manifestations, not life threatening.
- Class III – severe manifestations in which life is threatened, whose symptoms involve cardiogenic, respiratory and/or neurological failure.

## Results

**Fig. 2:**
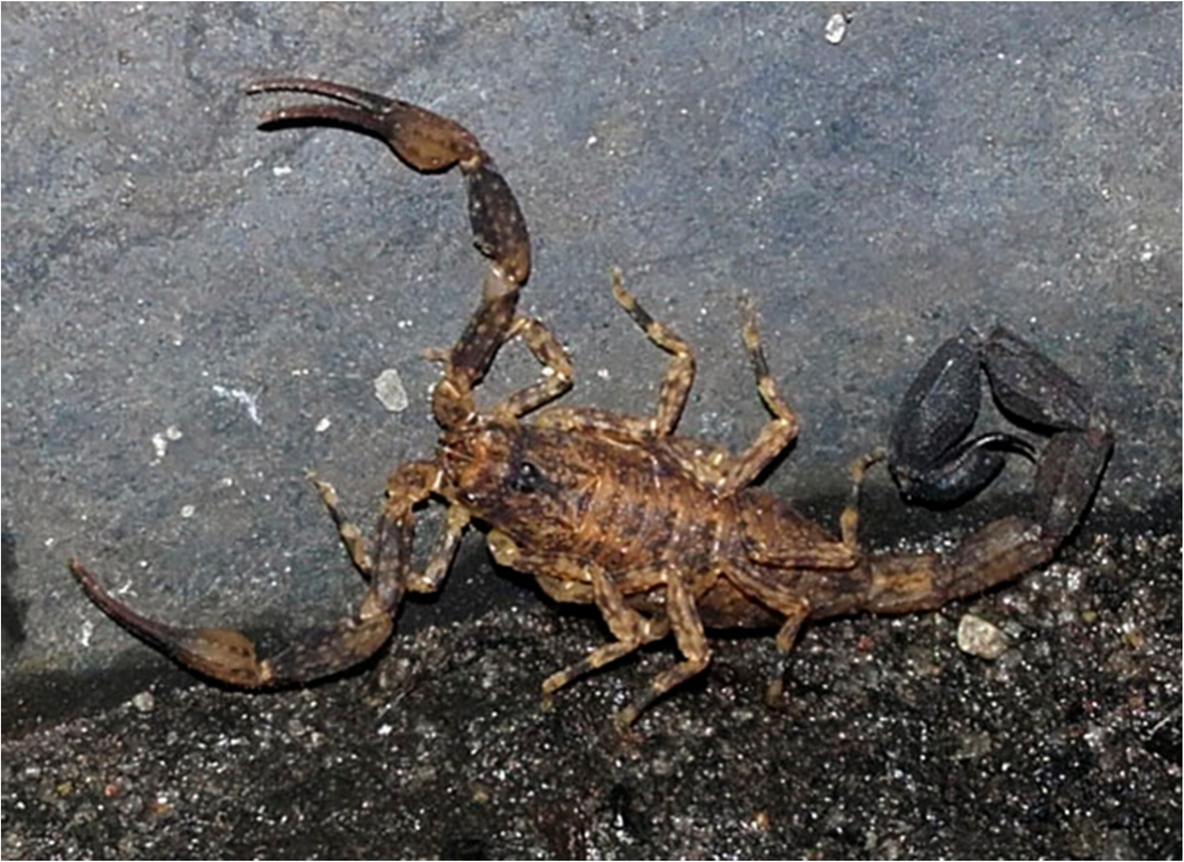
*Tityus silvestris* collected in the eastern Brazilian Amazon

Of the patients who were envenomed, seven (53.8 %) received medical assistance in Belém and six (46.2 %) in Ananindeua. The scorpions collected by the victims were identified as *T. silvestris* (Fig. 2), according to the taxonomic key of Lourenço [6]. All the scorpions were fixed with ethanol 70 % and are stored at the Laboratory of Medical Entomology and Venomous Animals in the Federal University of Pará.

The reported circumstances of the stings included: time of the day, environment and probable scorpion shelter (Table 1). In addition, other information considered relevant are shown in Table 2, including demographic data, age, gender, time elapsed from the envenomation to medical care and affected area of the body. In Table 3, local and systemic symptoms, severity parameters, clinical severity and treatment are presented.

**Table 1:** Circumstances of scorpionism by *T. silvestris* in Pará state, eastern Amazon, Brazil

Circumstances	No.	Percent
Time of the day		
Morning	8	61.6
Afternoon	3	23
Night	2	15.4
Environment		
Domiciliary	10	77
Extra-domiciliary	3	23
Scorpion shelter		
Garments	7	53.8
Fruits and vegetables	4	30.8
Debris	2	15.4

**Table 2:** Demographic profile of victims, elapsed time from envenomation to medical care and affected area of the body by *T. silvestris* stings in Pará state, eastern Amazon, Brazil

Variable	No.	Percent
Age group (years)	13	100.0
0-10	1	7.7
11-20	2	15.4
21-30	5	38.4
31-40	2	15.4
41-50	2	15.4
51-60	1	7.7
Md(IQR)^a^ 30(22–40)		
Gender	13	100.0
Male	7	53.8
Female	6	46.2
Time to medical care	13	100.0
≤2 h	10	77
2 h to 6 h	3	23
Sting site	13	100.0
Upper limbs	8	61.6
Lower limbs	3	23
Trunk	2	15.4

^a^Median and interquartle interval

**Table 3:** Clinical parameters of envenomation in victims of *T. silvestris* in Pará state, eastern Amazon, Brazil

Parameters	No.	Percent
Local symptoms		
ᅟPain	13	100.0
ᅟParesthesia	5	38.5
ᅟErythema	4	30.8
ᅟEdema	4	30.8
Systemic symptoms		
ᅟMalaise	1	7.7
ᅟNausea	2	15.4
ᅟVomiting	1	7.7
ᅟProstration	1	7.7
ᅟSomnolence	1	7.7
Severity of symptoms		
ᅟLocal manifestations	13	100.0
ᅟSystemic manifestations	3	23
Clinical severity		
ᅟClass I	10	77
ᅟClass II	3	23
Treatment		
ᅟAnalgesics	13	100.0
ᅟAntivenom	1	7.7

All patients with local manifestations were treated with analgesics and under clinical observation for 3 to 6 h. Out of the three Class II severity patients, only the one with malaise, nausea and prostration was treated with two ampoules of specific antivenom. Hematologic and biochemical assessments were not carried out and the clinical outcome of all patients were favorable. Each vial of antivenom contained 5 mL of product, and 1 mL of it neutralizes 1 mg of *T. serrulatus* venom in mice. This F(ab’)2 polyspecific hyperimmune equine antivenom was raised against *T. serrulatus* venom and produced by the Ezequiel Dias Foundation in Minas Gerais state, Brazil.

## Discussion

Epidemiological research conducted in the present study area indicate Belém and Ananindeua as the municipalities with the highest incidence of scorpionism in Pará state, being *T. obscurus* the main causative agent followed by *T. silvestris* [8, 9, 11, 12]. These two cities are the most populous of Pará state. The population density of Belém is 1,315 inhabitants/km^2^ whereas Ananindeua presents 2,477 inhabitants/km^2^ [10]. Since the number of confirmed envenomation provoked by *T. silvestris* was small in the present analysis, it is suggested a lower incidence of this species in the geographical areas of the study.

The species identified as *T. silvestris* (according to taxonomic features) belongs to the genus *Tityus* and the subgenus *Archaeotityus* [13]. It differs from other *Tityus* by its small body with scattered dark spots. In this species, there is distinct sexual dimorphism. Epidemiological reports of this species are only described by Asano et al. [9] and Martins et al. [8] in the studied region. Envenomation by other species of this subgenus, such as *T. pusillus*, were observed in Pernambuco state, Brazil [14].

Scorpionism was more frequent during daytime, corroborating the study Pardal et al. [12] in Pará state, which differs from the observation by Ribeiro et al. [15] in São Paulo state that did not find a period of the day with significant prevalence. Regarding the circumstances of envenomation, Maestri Neto et al. [11], in an epidemiological survey condeucted in Pará state, showed that the domiciliary environment had a higher incidence, which corroborates with the results of the present work. However, Santos et al. [16] in Minas Gerais, Brazil, showed that the work environment was the most affected.

It is known that scorpions are found in many different environments [5, 17]. The involved animals of the present study were found mostly in garments and vegetable leaves such as lettuce (*Lactuca sativa*) and fruits in clusters, such as peach palm (*Bactris gasipaes*), which when manipulated expose the scorpion to the person handling the plant. Scorpions have specific requirements regarding their habitat and the environmental conditions. Therefore, they can be found in modified environments, especially in urban areas [5]. Possani [17] stated that in Mexican cities scorpions are often found in public markets among fruits and vegetables.

According to the age of the victims, young adults were the group most affected by *T. silvestris* stings. The age range was 9–57 years old, with a median of 30 and interquartile range of 22–40 years. This variable was found in the Brazilian literature, with the age range between 20 and 49 years in the northern region of the country, in the states of Amazonas and Pará [4, 11, 18]. Regarding gender, men were slightly more prevailing.

The elapsed time between envenomation and hospital admission found in this study corroborates the findings of other studies in the Amazon region by Pardal et al. [12] and Queiroz et al. [18]. However, these observations did not agree with those of Chippaux [4], who described a longer time in the north than in other parts of Brazil. Probably the fastest hospital admission in the present case is due to the ease of transportation and better equipping of the health network in Belém and Ananindeua.

In this study, the upper limbs were the body area most affected by scorpion stings, which agrees with previous Brazilian studies concerning other scorpion species [4, 12, 18, 19]. This fact is probably associated with the use of the upper limbs for handling objects and execution of daily tasks.

Among the local symptoms of *T. silvestris* envenomation, pain at the sting site was the most common, followed by paresthesia, erythema and edema. These results are similar to those described for other Brazilian scorpions, both for the species of minor medical relevance – such as *T. pusillus* [14] and *Rhopalurus amazonicus* [20] – and the ones important to public health – as *T. obscurus*, *T. serrulatus, T. bahiensis* and *T. stigmurus* [12, 14, 20–23].

Systemic manifestations were reported in three victims of envenomation by *T. silvestris* aged between 22 and 57 years, classified in Class II of severity. A 22-year-old patient presented malaise, nausea and prostration. The other, a 48-year-old victim, had only one episode of vomiting, whereas the eldest patient, a 57-year-old person, had nausea and somnolence. These findings corroborate those found in the Brazilian literature, in which more severe envenomation usually affects patients younger than 15 years [24]. According to Reckziegel and Pinto [3], patients younger than 9 years have a higher risk of mortality.

*T. silvestris* is widely distributed in the Amazon region and responsible for mild cases of envenomation, particularly in Pará [5, 6]. To the best of our knowledge, this is the first report of systemic symptoms of envenomation by this species, which may indicate a potential aggressiveness of the venom. Previous reports by Martins et al. [8] and Asano et al. [9] in the same area describe cases of envenomation by scorpions in general, four and two cases, respectively, are attributable to *T. silvestris* with symptoms only at the sting site [8, 9].

Of the 13 cases of envenomation of the current study, only three were classified in Class II of severity. However, they showed symptoms of mild intensity. Of these, only the patient who developed symptoms of malaise, nausea and prostration received the specific antivenom, while the others were treated with symptomatic medications and life support. All patients had clinical improvement and were discharged from the hospital within six hours of admission. In Brazil, the treatment recommended by the Brazilian Ministry of Health for patients stung by scorpions depends on the severity of the case. For cases with signs and symptoms only at the sting site, symptomatic treatment and medical observation for 6 to 12 h is recommended, whereas Class II and Class III patients should receive specific antivenom [24].

## Conclusions

This study is the first to report the systemic symptomatology of envenomation by *T. silvestris* in the Brazilian Amazon, highlighting the medical relevance of this species in this region, whose systemic manifestations were of small magnitude, classified as Class II of severity. Research on the venom and clinical manifestations of the envenomation by the species should be performed to verify its real relevance to public health.

## References

[1] World Health Organization (2007). Rabies and envenomings: a neglected public health issue: report of a consultative meeting.

[2] Chippaux JP, Goyffon M (2008). Epidemiology of scorpionism: a global appraisal. Acta Trop.

[3] Reckziegel GC, Pinto VL (2014). Scorpionism in Brazil in the years 2000 to 2012. J Venom Anim Toxins incl Trop Dis.

[4] Chippaux JP (2015). Epidemiology of envenomations by terrestrial venomous animals in Brazil based on case reporting: from obvious facts to contingencies. J Venom Anim Toxins incl Trop Dis.

[5] Secretaria de Vigilância em Saúde (2009). Departamento de Vigilância Epidemiológica: Manual de Controle de Escorpiões, Série B.

[6] Lourenço WR (2002). Scorpions of Brazil.

[7] Khattabi A, Soulaymani-Bencheikh R, Achour S, Salmi LR (2011). Scorpion consensus expert group. Classification of clinical consequences of scorpion stings: consensus development. Trans R Soc Trop Med Hyg.

[8] Martins MA, Barradas L, Silva RHV, Pardal PPO (2002). Estudo clínico e epidemiológico dos acidentes por escorpião atendidos no Hospital Universitário João de Barros Barreto período de janeiro a dezembro de 1996. Rev Para Med.

[9] Asano ME, Arnund RM, Lopes FOB, Pardal JSO, Pardal PPO (1996). Estudo clínico e epidemiológico de 12 acidentes por escorpiões atendidos no Hospital Universitário João de Barros Barreto, Belém-Pará, no período de 1992–1995. Rev Soc Bras Med Trop.

[10] Instituto Brasileiro de Geografia e Estatística. Cidades: Pará Belém e Ananindeua; 2015. [http://cidades.ibge.gov.br/xtras/perfil.php?lang = &codmun = 150080&search = para|ananindeua]. Accessed on September 07, 2015.

[11] Maestri Neto A, Guedes AB, Carmo SF, Chalkidis HM, Coelho JS, Pardal PPO (2008). Aspectos do escorpionismo no Estado do Pará – Brasil. Rev Para Med.

[12] Pardal PP, Ishikawa EA, Vieira JL, Coelho JS, Dórea RC, Abati PA (2014). Clinical aspects of envenomation caused by *Tityus obscurus* (Gervais, 1843) in two distinct regions of Pará state, Brazilian Amazon basin: a prospective case series. J Venom Anim Toxins incl Trop Dis.

[13] Lourenço WR (2006). Nouvelle proposition de découpage sous-générique du genre *Tityus* C. L. Koch, 1836 (Scorpiones, Buthidae). Bol SEA.

[14] Albuquerque CMR, Porto TJ, Amorim MLP, Santana Neto PL (2009). Escorpionismo por *Tityus pusillus* Pocock, 1893 (Scorpiones; Buthidae) no Estado de Pernambuco. Rev Soc Bras Med Trop.

[15] Ribeiro AL, Rodrigues L, Jorge MT (2001). Aspectos clínicos e epidemiológicos do envenenamento por escorpiões em São Paulo e municípios próximos. Rev Patol Trop.

[16] Santos PLC, Martins FJ, Vieira RCPA, Ribeiro LC, Barreto BB, Leite ICG (2010). Características dos acidentes escorpiônicos em Juiz de Fora-MG. Rev APS.

[17] Possani LD (2005). El alacrán y su piquete.

[18] Queiroz AM, Sampaio VS, Mendonça I, Fé NF, Sachett J, Ferreira LCL, et al. Severity of scorpion stings in the Western Brazilian Amazon: a case–control study. Plos One. 2015;1–14. 10.1371/journal.pone.0128819 PMC4465172 26061734

[19] Guerra CMN, Carvalho LFA, Colosimo EA, Freire HBM (2008). Analysis of variables related to fatal outcomes of scorpion envenomation in children and adolescents in the State of Minas Gerais State, Brazil, from 2001 to 2005. J Pediatr.

[20] Fuentes-Silva D, Santos-Jr AP, Oliveira JS (2014). Envenomation caused by Rhopalurus amazonicus Lourenço, 1986 (Scorpiones, Buthidae) in Pará State, Brazil. J Venom Anim Toxins incl Trop Dis.

[21] Torrez PP, Quiroga MM, Abati PA, Mascheretti M, Costa WS, Campos LP (2015). Acute cerebellar dysfunction with neuromuscular manifestations after scorpionism presumably caused by Tityus obscurus in Santarém, Pará/Brazil. Toxicon.

[22] Bucaretchi F, Baracat ECE, Nogueira RJN, Chaves A, Zambrone FAD, Fonseca MRCC (1995). A comparative study of severe scorpion envenomation in children caused by Tityus bahiensis and Tityus serrulatus. Rev Inst Med Trop São Paulo.

[23] Lira-da-Silva RM, Amorim AM, Brazil TK (2000). Envenenamento por *Tityus stigmurus* (Scorpiones; Buthidae) no Estado da Bahia, Brasil. Rev Soc Bras Med Trop.

[24] Fundação Nacional de Saúde (BR) (2001). Manual de diagnóstico e tratamento de acidentes por animais peçonhentos.

